# Implementing an Educational Model for Cardiac Arrest Patients During Interfacility Transfers

**DOI:** 10.7759/cureus.79892

**Published:** 2025-03-01

**Authors:** Michael J Burla, Drew R York, Jeanne S Wishengrad, Peter C Michalakes, Holly A Stevens, Teresa L May

**Affiliations:** 1 Department of Emergency Medicine, MaineHealth Maine Medical Center Biddeford, Biddeford, USA; 2 Department of Emergency Medicine, Tufts University School of Medicine, Portland, USA; 3 College of Osteopathic Medicine, University of New England, Biddeford, USA; 4 Department of Research, MaineHealth Institute for Research, Scarborough, USA; 5 School of Medicine, Tufts University School of Medicine, Boston, USA; 6 Department of Emergency Medicine, Northern Light Mercy Hospital, Portland, USA; 7 Department of Pulmonary and Critical Care Medicine, MaineHealth Maine Medical Center Portland, Portland, USA; 8 Department of Medicine, Tufts University School of Medicine, Boston, USA

**Keywords:** emergency medical service, ems education, interfacility transfer, out of hospital cardiac arrest, post-rosc care

## Abstract

Background

Out-of-hospital cardiac arrest (OHCA) is a significant health concern in the United States, particularly in rural areas where patient care often depends on interfacility transfers (IFTs) to tertiary medical centers. In Maine, OHCA outcomes vary widely across regions, with no standardized education or protocols for the IFT of these patients. A key step toward improving care for this population is providing emergency medical service (EMS) personnel with education on essential aspects of post-return of spontaneous circulation (ROSC) management. To address this need, we developed a pilot virtual curriculum focused on post-ROSC care during IFT. The curriculum was designed to be virtual due to restrictions on in-person didactics at the time of implementation during the COVID-19 pandemic. This study aims to evaluate the feasibility of implementing a virtual curriculum that can be easily distributed. Additionally, we seek to analyze pre- and post-survey data from EMS personnel to assess changes in confidence, anxiety, and knowledge related to post-ROSC care.

Methods

Two local EMS departments, which handle the majority of IFTs for our institution, were invited to participate in the virtual educational model. The model consisted of a 27-minute recorded session divided into sections and distributed via an online platform in collaboration with Maine EMS. To assess the impact of the curriculum, EMS personnel completed electronic surveys administered through REDCap. A pre-survey was conducted before the curriculum, followed by a post-survey after its completion. The survey included questions measured on a 5-point Likert scale, covering confidence in resuscitation (five questions), anxiety during resuscitation (six questions), knowledge about post-ROSC care (eight questions), and attitudes toward the curriculum (three questions). Data analysis was performed using the Wilcoxon paired signed rank test to evaluate changes across these variables.

Results

A total of 18 EMS personnel participated in the curriculum (18/24; 75%), with all 18 completing the pre-survey and 10 completing the post-survey. Among those who completed both surveys, 70% agreed or strongly agreed that the IFT curriculum would improve patient care. Regarding knowledge of post-ROSC care, participants showed a mean improvement of +2.125 correct answers across the eight knowledge-based questions, with an average post-survey score of 6/8 (75%). However, there were no significant changes in confidence, skill, or anxiety levels, as indicated by a p-value of >0.9.

Conclusions

This pilot curriculum demonstrated the feasibility of delivering and completing a virtual educational model for EMS personnel on a topic not currently covered by Maine’s state protocols or existing educational programs. Pre-post survey results indicated some improvement in participants; knowledge of post-ROSC care, and 70% believed the curriculum would enhance patient care. Future efforts are underway to integrate this content as a permanent component of local EMS education, given the significant role of ROSC management during IFTs.

## Introduction

Out-of-hospital cardiac arrest (OHCA) remains one of the leading causes of death in the United States [1,2]. To improve outcomes for this high-risk population, the American Heart Association (AHA) emphasizes the importance of high-quality CPR and bystander-initiated CPR [3-5]. These interventions serve as the first steps in the on-scene management of OHCA patients. Once return of spontaneous circulation (ROSC) is achieved, care is typically continued by local emergency medical service (EMS) providers who transport the patient to the nearest ED. In rural areas, patients frequently present to small hospitals or isolated EDs, necessitating transfer to ICUs at larger tertiary care centers for definitive management. These interfacility transfers (IFTs), conducted by EMS providers, are often complicated by factors such as long distances, transport delays, and adverse weather conditions [6,7].

In Maine, EMS care is provided by a diverse network of small community departments with limited resources. Due to the state’s geographic characteristics, many rural hospitals initiate treatment before transferring patients via IFT, typically carried out by local EMS providers. Previous research has shown significant variation in OHCA outcomes across different regions and EMS departments [8-11]. Furthermore, studies indicate that EMS protocols specifically designed for OHCA patients can improve survival rates [10]. However, in Maine, all EMS agencies operate under the Maine EMS governing body, which provides only a basic OHCA protocol. While this protocol includes guidelines for basic and advanced life support, it lacks specific directives for post-ROSC care. Additionally, there is no standardized approach to physician orders or vital sign parameters for EMS providers, resulting in considerable variability in the management of hemodynamics, temperature, and ventilation during transport [12].

Recognizing these challenges, the AHA recently highlighted knowledge gaps in the care provided during IFT of ROSC patients, underscoring the need for a greater focus on this phase of care and the potential benefits of protocol-driven management [13]. These findings suggest an opportunity to improve OHCA outcomes by implementing a standardized curriculum for EMS agencies, specifically addressing post-ROSC care during IFT. This study aims to assess the feasibility of introducing such a curriculum to local EMS departments to enhance the quality of care delivered during IFT for OHCA patients.

## Materials and methods

Participants and recruitment

This prospective quantitative study involved EMS personnel completing a pre-survey (Appendix A) before viewing the curriculum, followed by a post-survey after enrollment. We collaborated with two EMS departments - Biddeford EMS and Northeast Ambulance Service - which manage the majority of IFTs for OHCA patients from MaineHealth Maine Medical Center Biddeford to MaineHealth Maine Medical Center Portland (60.9%; 14/23, in the last six months of 2021).

A total of 24 EMS staff members who regularly perform IFTs for this patient population were identified as eligible participants. To assess feasibility, a target participation rate of 70% was set, accounting for variations in staff availability [14]. The study population included basic trainees, emergency medical technicians (EMTs), advanced EMTs, and paramedics. Both EMS agency directors approved their staff’s participation in the pilot. Staff were informed of the study during general meetings, where it was emphasized that participation was voluntary and anonymous. Following these meetings, directors emailed staff with details on consent and a link to access the curriculum. Survey completion remained optional.

Enrollment

Participants enrolled through the Maine EMS protocols website, a widely used educational hub accessible to all Maine EMS personnel and commonly utilized for continuing education hours. Consent was implied upon reading the study description and submitting the surveys.

Curriculum

The curriculum was developed based on AHA guidelines and relevant literature on post-ROSC care. It was created by the principal investigator in collaboration with a fellowship-trained EMS director (Appendix B). The curriculum objectives were determined through discussions with local EMS departments to identify the most relevant topics for post-ROSC care during IFT.

The content was recorded and edited using Camtasia software (TechSmith Corporation, East Lansing, MI, USA) and presented as a PowerPoint (Microsoft Corporation, Redmond, WA, USA) file. Participants viewed slides accompanied by a pre-recorded script. Key components of the curriculum are outlined in Table 1.

**Table 1:** Key components of the curriculum

Parameter	Target/medications
Blood pressure management	Maintain mean arterial pressure >80 mmHg
Oxygen target	SpO₂ 92-98%
Temperature target	<37°C (goal: 33-36°C)
Glucose target	80-180 mg/dL
Sedation	Propofol, fentanyl, and remifentanil
Vasoactive medications	Norepinephrine, epinephrine, and vasopressin
Paralytics	Vecuronium and cisatracurium
Cerebral edema management	Mannitol

Data collection

Pre- and post-surveys were anonymous and administered electronically through a HIPAA-compliant MaineHealth REDCap [15] database, accessible only to the study team via a password-protected login. No HIPAA-protected information was collected.

The 20-question survey was developed by the principal investigator and reviewed by the institution’s EMS director. Question validation was based on the objectives established during initial discussions with EMS departments. The survey assessed participants’ knowledge of post-ROSC care, as well as their perceived skill, comfort level, and confidence. Specifically, it included eight multiple-choice questions covering targeted temperature management, post-ROSC blood pressure management, factors influencing neurological outcomes, and medications used in post-ROSC care. Knowledge scores were calculated as the proportion of correct responses.

Additionally, the survey included questions measuring perceived skill, comfort level (anxiety), and confidence, each assessed on a 5-point Likert scale. Scores ranged from 1 to 5 for skill, up to 25 for confidence, and up to 30 for anxiety, with a maximum total score of 60. Pre- and post-surveys were administered approximately one month apart.

Data analysis

As this was a pilot study of a new curriculum with no preliminary data available for power analysis, all analyses were exploratory, with no expectation of statistical significance. Survey data were summarized using descriptive statistics, with categorical data reported as frequencies. The Wilcoxon paired signed rank test was used for categorical data analysis.

## Results

A total of 18 out of 24 (75%) eligible EMS personnel from local EMS agencies participated in the curriculum and completed the pre-survey. The baseline demographics of the participants are summarized in Table 2. The majority were male (67%). Notably, 40% of participants reported having no current Advanced Cardiovascular Life Support certification, while 50% had undergone training within the past two years, and 10% had completed training more than two years ago.

**Table 2:** Pre-survey demographics ACLS, Advanced Cardiac Life Support; EMT, emergency medical technician

Characteristic	N = 18 (%)
Age	
>50 years	9 (50%)
21-30 years	5 (28%)
31-40 years	1 (5.6%)
41-50 years	3 (17%)
Gender	
Female	4 (22%)
Male	12 (67%)
Other	2 (11%)
Most recent ACLS training	
No current ACLS training	9 (50%)
0-6 months ago	1 (5.6%)
6-12 months ago	2 (11%)
12-18 months ago	4 (22%)
19-24 months ago	1 (5.6%)
>24 months ago	1 (5.6%)
Current level of training	
Advanced EMT	5 (28%)
Critical care paramedic	2 (11%)
EMT	5 (28%)
Paramedic	6 (33%)

Of the 18 participants, 10 completed both the pre- and post-surveys. Those who completed the post-survey had a slightly higher proportion of male participants compared to those who did not (80% vs. 67%). The distribution of training levels was similar between both groups (advanced EMT: 28% vs. 30%; critical care paramedic: 11% vs. 20%; EMT: 28% vs. 20%; paramedic: 33% vs. 30%). Results for questions using the 4-point Likert scale are presented in Table 3. There was no statistically significant difference between pre- and post-survey results regarding confidence and anxiety across various clinical scenarios.

**Table 3:** Pre- and post-survey results using the Likert Scale

Characteristic	Pre (N = 10)	Post (N = 10)	p-Value
Confidence level in the following scenarios			
Leading a resuscitation	-	-	>0.9
1 Not confident	0 (0%)	0 (0%)	-
2 Somewhat confident	4 (40%)	5 (50%)	-
3 Confident	4 (40%)	3 (30%)	-
4 Very confident	2 (20%)	2 (20%)	-
Working with a team	-	-	>0.9
1 Not confident	0 (0%)	0 (0%)	-
2 Somewhat confident	1 (10%)	1 (10%)	-
3 Confident	6 (60%)	5 (50%)	-
4 Very confident	3 (30%)	4 (40%)	-
Recognizing when to seek help	-	-	>0.9
1 Not confident	0 (0%)	0 (0%)	-
2 Somewhat confident	1 (10%)	0 (0%)	-
3 Confident	5 (50%)	7 (70%)	-
4 Very confident	4 (40%)	3 (30%)	-
Ability to supervise team members	-	-	0.8
1 Not confident	1 (10%)	1 (10%)	-
2 Somewhat confident	2 (20%)	4 (40%)	-
3 Confident	5 (50%)	3 (30%)	-
4 Very confident	2 (20%)	2 (20%)	-
Knowing how to obtain additional help	-	-	>0.9
1 Not confident	0 (0%)	0 (0%)	-
2 Somewhat confident	2 (20%)	2 (20%)	-
3 Confident	5 (50%)	5 (50%)	-
4 Very confident	3 (30%)	3 (30%)	-
Anxiety level when performing these skills			
Chest compression	-	-	>0.9
1 Not anxious	9 (90%)	9 (90%)	-
2 Somewhat anxious	1 (10%)	1 (10%)	-
3 Anxious	0 (0%)	0 (0%)	-
4 Very anxious	0 (0%)	0 (0%)	-
Airway management	-	-	0.8
1 Not anxious	4 (40%)	4 (40%)	-
2 Somewhat anxious	4 (40%)	5 (50%)	-
3 Anxious	2 (20%)	1 (10%)	-
4 Very anxious	0 (0%)	0 (0%)	-
Recognizing different cardiac arrhythmias	-	-	0.8
1 Not anxious	7 (70%)	5 (50%)	-
2 Somewhat anxious	2 (20%)	5 (50%)	-
3 Anxious	1 (10%)	0 (0%)	-
4 Very anxious	0 (0%)	0 (0%)	-
Choosing synchronized cardioversion or defibrillation	-	-	>0.9
1 Not anxious	8 (80%)	8 (80%)	-
2 Somewhat anxious	0 (0%)	0 (0%)	-
3 Anxious	1 (10%)	1 (10%)	-
4 Very anxious	1 (10%)	1 (10%)	-
Performing adequate chest compressions	-	-	>0.9
1 Not anxious	9 (90%)	9 (90%)	-
2 Somewhat anxious	1 (10%)	1 (10%)	-
3 Anxious	0 (0%)	0 (0%)	-
4 Very anxious	0 (0%)	0 (0%)	-
Intraosseous line placement	-	-	>0.9
1 Not anxious	4 (40%)	5 (50%)	-
2 Somewhat anxious	5 (50%)	5 (50%)	-
3 Anxious	1 (10%)	0 (0%)	-
4 Very anxious	0 (0%)	0 (0%)	-

Results for the knowledge-based questions and participant feedback are summarized in Table 4. Most questions showed an improvement in correct responses from pre- to post-survey, with an average increase of +2.125 correct answers (75% of the questions). However, these changes were not statistically significant. Additionally, 80% of participants indicated that feedback following a resuscitation was often or very often necessary, while 70% agreed or strongly agreed that the curriculum would enhance patient care.

**Table 4:** Pre and post questions on knowledge and feedback ^*^ Correct answers for the knowledge-based questions are highlighted in bold. Answer choices (A, B, C, and D) represent different response options for each question. For the complete survey, please refer to Appendix A. ROSC, return of spontaneous circulation

Questions^*^	Pre (N = 10)	Post (N = 10)	p-Value
Why is targeted temperature management important in post-ROSC care?	-	-	>0.9
A. Targeted temperature management has been shown to improve neurologic outcomes in post cardiac arrest patients.	7 (70%)	6 (60%)	-
B. Temperature management has been shown to improve blood glucose levels.	3 (30%)	4 (40%)	-
C. Targeted temperature management has been shown to improve survival rates in post-cardiac arrest patients.	0 (0%)	0 (0%)	-
D. Temperature management has been shown to improve blood pressure.	0 (0%)	0 (0%)	-
What temperature is generally safe for post-ROSC care?	-	-	0.3
A. 22-26°C	0 (0%)	0 (0%)	-
B. 28-32°C	2 (20%)	0 (0%)	-
C. 32-36°C	8 (80%)	10 (100%)	-
D. 36-40°C	0 (0%)	0 (0%)	-
What is the importance of strict glucose management?	-	-	0.1
A. Strict glucose management leads to better survival rates.	5 (50%)	4 (40%)	-
B. Persistent hyperglycemia has been shown to lead to an increase in mortality.	4 (40%)	1 (10%)	-
C. The ideal glucose range post-ROSC is 100-140 mg/dL.	0 (0%)	1 (10%)	-
D. Strict glucose management has not been shown to change mortality.	1 (10%)	4 (40%)	-
What is the importance of blood pressure management?	-	-	0.4
A. Strict blood pressure management has not been shown to change mortality.	1 (10%)	0 (0%)	-
B. A systolic blood pressure of less than 90 mmHg or less than 100 mmHg is associated with high mortality.	6 (60%)	8 (80%)	-
C. Hypotension and hypertension have the same rates of mortality with post-ROSC care.	2 (20%)	0 (0%)	-
D. A systolic blood pressure less than 90 mmHg or less than 100 mmHg is associated with low mortality	1 (10%)	2 (20%)	-
What is the ideal blood pressure in post-ROSC care?	-	-	0.2
A. Avoiding hypertension during post-ROSC care is most ideal.	0 (0%)	0 (0%)	-
B. Achieving a MAP above 80 during post-ROSC care is most ideal.	0 (0%)	4 (40%)	-
C. Achieving a MAP above 65 during post-ROSC care is most ideal.	8 (80%)	4 (40%)	-
D. Avoiding hypotension during post-ROSC care is most ideal.	2 (20%)	2 (20%)	-
What role does paralysis play in post-ROSC care?	-	-	0.4
A. Paralysis plays no role in the care of post-ROSC patients aside from rapid sequence intubation.	1 (10%)	0 (0%)	-
B. Paralysis can help prevent shivering, which can have an adverse impact on temperature management.	5 (50%)	8 (80%)	-
C. Paralysis has been shown to improve neurologic outcomes.	3 (30%)	2 (20%)	-
D. Paralysis can help prevent hypertension, which can have an adverse effect on glucose management.	1 (10%)	0 (0%)	-
What is the best time for prognostication in post-ROSC patients?	-	-	0.2
A. Immediately after achieving ROSC	1 (10%)	0 (0%)	-
B. After a minimum of 24 hours after ROSC	4 (40%)	0 (0%)	-
C. After a minimum of 72 hours after ROSC	2 (20%)	10 (100%)	-
D. Once the patient is rewarmed to normothermia	3 (30%)	0 (0%)	-
How often is feedback or debriefing needed after resuscitation?	-	-	>0.9
1 Very rarely	0 (0%)	0 (0%)	-
2 Rarely	1 (10%)	1 (10%)	-
3 Sometimes	1 (10%)	1 (10%)	-
4 Often	3 (30%)	2 (20%)	-
Very often	5 (50%)	6 (60%)	-
Do you believe this curriculum will improve patient care?	-	-	>0.9
1 Strongly disagree	1 (10%)	1 (10%)	-
2 Disagree	0 (0%)	0 (0%)	-
3 Neither agree nor disagree	2 (20%)	2 (20%)	-
4 Agree	3 (30%)	3 (30%)	-
5 Strongly agree	4 (40%)	4 (40%)	-

## Discussion

In this feasibility study, we aimed to develop and implement a virtual EMS curriculum focused on IFT for post-ROSC patients. We successfully recruited the majority of EMS providers from two pilot departments responsible for the IFT of ROSC patients. While 75% of knowledge-based questions on the post-survey showed improvement from incorrect to correct answers, this change was not statistically significant. However, the study was not powered to assess knowledge retention, as the primary focus was feasibility. Notably, 70% of participants agreed or strongly agreed that the IFT curriculum would improve patient care. These findings highlight the potential for broader dissemination to other community EMS departments to enhance knowledge of post-ROSC care during IFT.

There is limited existing literature on the care provided during IFT following cardiac arrest [16-18], and most available studies do not focus on predominantly rural populations. Our previous research found that the majority of OHCA patient IFTs in Maine were performed by local community EMS departments, often with little to no guidance on management [12]. The combination of potential knowledge deficits and the heavy reliance on IFT in rural areas, such as Maine, could have significant implications for post-ROSC care and OHCA survival. Studies have already demonstrated that rural populations experience lower rates of bystander CPR and defibrillator use [19-22]. Combined with longer transport times [6,7] and increased reliance on IFT for definitive care, patients in rural regions face an elevated risk of poor OHCA outcomes [6,7]. As noted in the introduction, the AHA recognizes knowledge gaps in the care provided during IFT of OHCA patients [13]. Addressing these gaps is crucial to improving post-ROSC care and patient outcomes. Implementing our curriculum has the potential to fill this educational void, ultimately contributing to reduced morbidity and mortality.

Community EMS educational outreach has been shown to improve patient care [23]. While EMS-focused education is not a new concept, there are currently no well-established models for managing OHCA patients during IFT. Most local EMS departments are not structured to handle the transport of critically ill patients, which likely explains why IFT management is not explicitly addressed in Maine’s statewide protocols. However, this is a scenario that many community EMS departments routinely encounter. Our curriculum was originally designed as a virtual program in response to the COVID-19 pandemic, when in-person training was not feasible. Feedback from participants indicated a strong interest in incorporating an in-person component, which we plan to implement in the future to encourage greater participation. Additionally, Maine EMS has expressed openness to updating statewide protocols to address the management of critically ill patients during IFT. We also plan to introduce a standardized physician order form specifically for OHCA patients during IFT to ensure adherence to protocols. Many aspects of patient management can be standardized through protocol-driven care [13], which could then be adapted and expanded to other rural regions across the country. We believe that empowering community EMS departments in rural areas with enhanced knowledge and training will have far-reaching benefits for public health.

Limitations

This study has several limitations. The survey questions were designed based on knowledge gaps identified by local EMS departments and the EMS director, rather than validated assessment tools. However, this approach was necessary given the lack of existing literature on EMS education for IFT of ROSC patients. Additionally, the study’s reliance on EMS personnel participation introduces the risk of low engagement, which could impact the results. Successful assessment of the pilot program’s feasibility depends on active involvement, and this limitation was evident, as 18 EMS personnel participated in the curriculum, but only 10 completed both the pre- and post-surveys.

Similarly, limited engagement could affect the study’s power, even though statistical significance was not the primary expectation. A smaller sample size may reduce the accuracy of measuring changes in knowledge, skills, confidence, and anxiety, making it more difficult to generalize findings to other EMS personnel and agencies - especially in rural regions that may face challenges distinct from those in Maine. Our long-term goal is to refine the curriculum to better suit the needs of Maine and other rural areas where IFT of ROSC patients is common.

We recognize that a larger sample size would be necessary to strengthen the findings and address these limitations. However, this study was primarily designed to evaluate program feasibility during a period when in-person gatherings were restricted.

## Conclusions

Our study demonstrated the feasibility of implementing a virtual curriculum focused on managing OHCA patients during IFT. This pilot curriculum addressed a critical gap not covered by statewide protocols, despite being a scenario frequently encountered by local EMS departments. While the study was underpowered to detect statistically significant differences in knowledge-based questions, most pre- and post-survey responses showed an increase in the selection of correct answers. Additionally, the majority of participants believed that the curriculum would improve patient care. Given the lack of standardized guidelines for managing OHCA patients during IFT, future efforts are underway to expand the curriculum and develop protocol-driven care.

## Appendices

Appendix A

ROSC Interfacility Transfer Pre-curriculum Survey

Maine EMS ROSC interfacility transfer survey: Before beginning the curriculum, the research team asks that you complete this pre-curriculum survey. The purpose of this survey is to gauge your current background, knowledge, and comfort level with post cardiac arrest patients. Participation is completely voluntary, and you may choose to skip any question(s) that you do not wish to answer.

Have you completed the EMS curriculum on interfacility transfer of ROSC patients?

 Yes

 No

1. What EMS department are you primarily affiliated with?

 Biddeford EMS

 NorthEast EMS

2. What is your age?

 <20 years old

 21-30 years old

 31-40 years old

 41-50 years old

 >50 years old

3. What is your gender?

 Female

 Male

 Other

 Decline to respond

4. When was your most recent Advanced Cardiac Life Support (ACLS) training successfully completed?

 No current ACLS training

 0- 6 months ago

 6-12 months ago

 12-18 months ago

 >18 months ago

 18-24 months ago

 > 4 months ago

5. What is your current level of training?

 Emergency medical responder (EMR)

 Emergency medical technician (EMT)

 Advanced EMT (AEMT)

 Paramedic

 Critical care paramedic

 None of the above

 Other (please specify)

The following section includes questions about your skill in and confidence with managing IFT for a cardiac arrest patient.

6. On a scale of 1 to 5, where 1 = unskilled and 5 = skilled, how would you rate your current level of skill in life saving techniques for cardiac arrest patients?

1

2

3

4

5

7. Please rate your confidence level in the following areas during patient resuscitation:

Not confident

Somewhat confident

Confident

Very confident

7.1 Leading a resuscitation

7.2 Working with a team to handle a resuscitation

7.3 Recognizing when to get additional help during a resuscitation

7.4 Ability to supervise team members during a resuscitation

7.5 Knowing how to get additional help during a resuscitation

8. Please rate your anxiety level when performing the following skills during patient resuscitation:

Not anxious

Somewhat anxious

Anxious

Very anxious

8.1 Airway management

8.2 Chest compressions

8.3 Recognizing different cardiac arrhythmias

8.4 Choosing synchronized cardioversion or defibrillation

8.5 Adequate chest compressions

8.6 Intraosseous line placement

9. In how many resuscitation events (a real critically ill patient in the field with or without arrest) have you participated in the last two years?

 ≤5

 6-10

 11-15

 16-20

 >20

9.1 In how many resuscitation events (total, including simulation) have you participated in the last two years?

 ≤5

 6-10

 11-15

 16-20

 >20

10. Please rate how frequently you have been involved in the following types of resuscitation cases in the last two years:

Never (0%)

Rarely/seen a few (<10%)

Sometimes/but have heard reports of many more (10-50%)

Often/seen many cases first hand (50-75%)

Very often/about once a week (>75%)

10.1 Cardiac arrest in the field

10.2 Inter facility transfer of post cardiac arrest patient

10.3 Inter facility transfer of unstable STEMI

10.4 Any patient with unstable vitals from the field

The following section includes questions about your knowledge in caring for a cardiac arrest patient after the return of spontaneous circulation (ROSC).

11. Why is targeted temperature management important in post return of spontaneous circulation (ROSC) care?

 A.  Targeted temperature management has been shown to improve neurologic outcomes in post cardiac arrest patients.

 B.  Temperature management has been shown to improve blood glucose levels.

 C.  Targeted temperature management has been shown to improve survival rates in post cardiac arrest patients.

 D.  Temperature management has been shown to improve blood pressure.

12. What temperature is generally safe for post-ROSC care?

 A.  22-26°C

 B.  28-32°C

 C.  32-36°C

 D.  36-40°C

13. What is the importance of strict glucose management in post-ROSC care?

 A.  Strict glucose management leads to better survival rates.

 B.  Persistent hyperglycemia has been shown to lead to an increase in mortality.

 C. The ideal glucose range post-ROSC is 100-140 mg/dL.

 D.  Strict glucose management has not been shown to change mortality.

14. What is the importance of strict blood pressure management in post-ROSC care?

 A.  Strict blood pressure management has not been shown to change mortality.

 B.  A systolic blood pressure less than 90 mmHg or less than 100 mmHG is associated with high mortality.

 C.   Hypotension and hypertension have the same rates of mortality with post-ROSC care.

 D.  A systolic blood pressure less than 90 mmHg or less than 100 mmHg is associated with low mortality.

15. What is the ideal blood pressure in post-ROSC care?

 A.  Avoiding hypertension during pos- ROSC care is most ideal.

 B.  Achieving a mean arterial pressure (MAP) above 80 during post-ROSC care is most ideal.

 C.  Achieving a mean arterial pressure (MAP) above 65 during post-ROSC care is most ideal.

 D.  Avoiding hypotension during post-ROSC care is most ideal.

16. What role does paralysis play in the care of a post-ROSC patient?

 A.  Paralysis plays no role in the care of post-ROSC patients aside from rapid sequence intubation.

 B.  Paralysis can help prevent shivering, which can have an adverse impact on temperature management.

 C.  Paralysis has been shown to improve neurologic outcomes.

 D.  Paralysis can help prevent hypertension, which can have an adverse effect on glucose management.

17. When is the best time for prognostication in a post-ROSC patient?

 A. Immediately after achieving ROSC

 B. After a minimum of 24 hours after ROSC

 C. After a minimum of 72 hours after ROSC

 D. Once the patient is rewarmed to normothermia

The following section includes questions about your opinion on post resuscitation feedback and improving the care of cardiac arrest patients during interfacility transfer.

18. How often is feedback and debriefing from colleagues following a resuscitation event necessary?

Very rarely/typically not necessary

Rarely/only for unique scenarios

Sometimes/depends on the case

Often/can learn something from most cases

Very often/every case

19. Curriculum and a protocol specifically designed for interfacility transfer of cardiac arrest patients will improve care delivered to this patient population.

Strongly disagree

Disagree

Neither agree nor disagree

Agree

Strongly agree

20. Please describe if there is anything else you think would improve the care of this patient population.

Appendix B
